# Fungus Hæmatodes in Cattle and Horses, with Notes on Cases in Practice*Read before the Pennsylvania State Veterinary Association.

**Published:** 1893-03

**Authors:** James A. Waugh

**Affiliations:** Allegheny, Pa.


### FUNGUS HÆMATODES IN CATTLE AND HORSES, WITH NOTES ON CASES IN PRACTICE.*

BY JAMES A. WAUGH, V.S., Allegheny, Pa.

Fungus hæmatodes, or encephaloid cancer, affects the orbit and contiguous parts, and occurs occasionally in cattle, horses and all classes of domesticated animals in all parts of our country. However, it is seen most frequently in the warm climate of the Southern States, and more especially in the Rocky Mountain country and in the States and Territories bordering on Old Mexico. A positive diagnosis may prove exceedingly difficult during the early stages of this disease. The first symptoms observed are usually an inflamed and watery condition of one eye, with evidence of considerable pain manifested by the patient being uneasy and elevating and depressing the head occasionally, and at other times stand-

* Read before the Pennsylvania State Veterinary Association.

ing with the feet widely apart, and drooping the eyelids and hanging the head. There is considerable pain, which causes sleeplessness and gradual loss of condition. The owner, or attending veterinarian, will readily realize that the patient is suffering from something more serious than opthalmia, and will likely examine the teeth for caries and the ears for parasites, but will find no evidence of disease in these organs. Ordinary opthalmic remedies may palliate the suffering and temporarily mitigate the virulence of the disease; yet it will progress gradually and finally develop into a cancerous tumor with an irregular surface, which bleeds readily on irritation or excoriation, and emits a characteristic odor which is very offensive, especially during warm weather. The tumor may involve any part of the orbit or contiguous parts, and sometimes the contents of the foramina are affected beyond the reach of the surgeon's scalpel. I have observed that this disease develops very slowly during the early, and very rapidly during the later stages of its existence. The alteration in the eye, and surrounding structures, may be only very slight or very extensive; evidently much depends upon the length of time and the malignancy of the disease. These tumors are a source of much annoyance to the unfortunate patients, and cause so much suffering that the animals frequently rub the affected parts against obstructions, which causes free hemorrhage and large granular sores.

*Causes.*—Severe and neglected opthalmia; accidental local injuries due to shipping in cars or other modes of transportation; play or quarrels among live stock; stings of insects or reptiles; continuous exposure to rays of hot sun; change of climate; drinking water charged with an excess of alkaline salts; also exposure to piercing winds and sand storms. A cancerous diathesis appears to exist and develop in certain families of mankind and the lower animals, which is well worthy of investigation and further study.

*Treatment.*—Cast and secure the patient by proper arrangements, and administer local or general anæsthetics. Early extirpation of any tumor or tumors that may develop on the inner surface of the eyelids or canthus, or on the membrani nictitans, and include a small amount of the healthy with the diseased tissues in order to insure complete removal of the cause of the trouble. Dress the wounds with modern astringent and antiseptic remedies, and apply proper dressings and bandages. These mild cases generally recover after this form of treatment. The eyeball is frequently involved in the diseased process and must be extirpated with all surrounding tissues included in the orbital cavity; then apply a heated bulb cautery iron to the surfaces of the wound; pack the cavity with marine lint or absorbent cotton, saturated in some antiseptic lotion; dress with bandages. Remove the lint or cotton in about fifteen or eighteen hours; then wash twice daily and dress with astringent and antiseptic lotions; cover lightly with cotton cloth to prevent annoyance from dust or foreign substances and insects. Feed moderately and exercise regularly during convalescence. I treated a case in which the tumor had evidently developed from the posterior part and periosteum of its wall, which caused degeneration and destruction of the walls of the orbit, and the main body of the tumor was forced into and lodged in the frontal and superior maxillary sinuses on the affected side of the face. Trephining was necessary in the treatment of this case, which was very interesting and will be mentioned in my notes. There may be alarming and extensive hemorrhage during the operation, but this should not deter the surgeon from completing the operation in a thoroughly scientific manner as described.

CASE NOTES.

No. 1. Large black mare, age seven years, and in good flesh, was brought to our place during the autumn of 1882. This was the first case of fungus hæmatodes that I had seen in the horse, and my brother—W. J. Waugh—and myself had just obtained our veterinary degrees that year, and had a penchant for surgical operations. We cast and secured the patient with double side-lines; then removed from the right costal region a tumor about the size of a walnut; also from the middle part of upper lip a tumor about the size of a hickory-nut. We next turned our attention to a very large and unsightly tumor, involving one entire orbit and surrounding tissues, which, when removed, weighed a trifle over four pounds. We passed crucial sutures through the main body of the tumor and knotted loops, which were used instead of tenaculums, then dissected the diseased skin away from the side of the face and used a curved bistoury for removing the diseased mass from the orbital cavity. We were very careful to observe Prof. Laws' advice to remove, as far as possible, all the diseased tissues, but we concluded to use only the potential cautery, then dressed the wounds with astringent and antiseptic lotions, and filled the orbital cavity with marine lint, secured with sutures and bandages. The packing and dressings were removed in fifteen hours, then the wounds were washed and dressed twice daily. The patient convalesced nicely for about forty days, and was then returned for further treatment, as the tumor had reappeared and refilled the orbital cavity. We then resorted to the actual cautery, followed by proper dressings. The patient recovered and remained apparently sound for over two years and raised two good colts, and has been used as a brood mare up to the present time. The tumor fills the orbital cavity about once every two years, then a country medical doctor resorts to the treatment which proved successful with us. The colts have remained healthy to date.

No. 2. Ordinary and middle-aged cow, affected with fungus hæmatodes of the inner surface of the upper eyelid. Treatment: Same as recommended in article. Result: Permanent recovery.

No. 3. Medium-sized draft mule, affected with small fungus hæmatodes tumor about size of walnut, protruding over the eyeball and attached to the inner surface of the upper eyelid. Treatment: Same as mentioned in article. Result: Complete recovery.

No. 4. Draft mule affected with fungus hæmatodes, involving the membrani nictitans. Treatment: Removal of the diseased mass as indicated in article. Result: Rapid recovery and return to usual work.

No. 5. Cavalry horse affected with fungus hæmatodes tumor about the size of a hazel-nut growing from inner surface of lower eyelid. Treatment: Removal of V-shaped mass; wire-sutured the wound and dressed as usual in similar cases. Result: Rapid recovery.

No. 6. Cavalry horse affected with inflamed eye when received at military post in New Mexico. I supposed this horse had been injured in shipping. Treatment resulted in marked improvement, and it was assigned to duty to a camp in the field. It was soon returned for treatment. The patient evinced signs of suffering from much pain, and I presumed the trouble was probably due to the sting of some insect, or the rays of hot sun, or exposure to sand storms. The patient was treated occasionally, and kept under observation for about two years. I finally diagnosed the trouble to be a neuromatous tumor affecting the nerves of the eye, and recommended the horse to be condemned and sold. I saw and operated on this horse some eighteen months afterwards in southern New Mexico, and found the fungus hæmatodes tumor located in the frontal and superior maxillary sinuses, as stated in article. Result: The weather was extremely hot, and the treatment proved so tedious and disagreeable that the owner became discouraged and destroyed the animal.

No. 7. The writer and his student, C. W. Boyd, were invited to see a case of fungus hæmatodes in an aged draft horse in a young veterinarian's infirmary. The tumor was situated in the back part of the orbital cavity, and pushed the eye almost beyond and through the eyelids. A young medical doctor extirpated the eyeball very dexterously, but he and the young veterinarian hesitated about attempting removal of the main body of the tumor which was the real source of the trouble; but I urged the importance and necessity of a complete operation. The doctor then cut into the tumor, and the hemorrhage was alarming and extensive, and they feared it would be impossible to arrest the hemmorrhage if the operation was finished. The orbital cavity was firmly plugged with absorbent cotton saturated in Monsel's solution, and the eyelids were securely sutured. The hemorrhage continued and caused rupture of the connective tissue under the skin for considerable space around the orbit. Bandages were firmly applied and secured to limit the hemorrhage. The patient was returned home in a few days. I was called to see this patient eight days afterwards, and was greatly surprised at finding the bandages intact and exactly the same as I had seen them applied at the operation. A slough about the size of an ordinary table-plate came away on removal of the bandages. The wound was rapidly dressed with carbolized lotion, and the owner was recommended to consult the attending veterinarian about final disposal of the subject. The extensive and long-continued pressure evidently destroyed nearly all of the malignant growth, and the treatment was continued, and the animal improved and almost recovered in about three months; but, unfortunately, it suffered from an attack of paralysis and succumbed to the disease.

Dr. N. Rectenwald has a very interesting case under treatment, which we will report at an early date.

